# Salmon Bias or Red Herring? Comparing Adult Mortality Risks (Ages 30–90) between Natives and Internal Migrants: Stayers, Returnees and Movers in Rotterdam, the Netherlands, 1850–1940

**DOI:** 10.1007/s12110-017-9303-1

**Published:** 2017-10-17

**Authors:** Paul Puschmann, Robyn Donrovich, Koen Matthijs

**Affiliations:** 1 Radboud Group for Historical Demography and Family History, Department of History, Radboud University, Erasmusplein 1, 6525 HT Nijmegen, the Netherlands; 2 Family and Population Studies, Centre for Sociological Research, KU Leuven, Parkstraat 45, 3000 Leuven, Belgium

**Keywords:** Healthy migrant effect, Salmon bias, Migration, Mortality, Rotterdam, The Netherlands

## Abstract

The purpose of this research is to empirically test the salmon bias hypothesis, which states that the “healthy migrant” effect—referring to a situation in which migrants enjoy lower mortality risks than natives—is caused by selective return-migration of the weak, sick, and elderly. Using a unique longitudinal micro-level database—the Historical Sample of the Netherlands—we tracked the life courses of internal migrants after they had left the city of Rotterdam, which allowed us to compare mortality risks of stayers, returnees, and movers using survival analysis for the study group as a whole, and also for men and women separately. Although migrants who stayed in the receiving society had significantly higher mortality risks than natives, no significant difference was found for migrants who returned to their municipality of birth (returnees). By contrast, migrants who left for another destination (movers) had much lower mortality risks than natives. Natives who left Rotterdam also had significantly lower mortality risks than natives who stayed in Rotterdam. Female migrants, in particular, who stayed in the receiving urban society paid a long-term health price. In the case of Rotterdam, the salmon bias hypothesis can be rejected because the lower mortality effect among migrants was not caused by selective return-migration. The healthy migrant effect is real and due to a positive selection effect: Healthier people are more likely to migrate.

**Electronic supplementary material:**

The online version of this article (10.1007/s12110-017-9303-1) contains supplementary material, which is available to authorized users.

Many European and North American studies report that migrants have lower mortality risks than the native-born population (Alter and Oris 2005; Markides and Eschbach 2005). This so-called healthy migrant effect is found in both contemporary and historical settings, and it has been explained by differences in early life conditions (Alter and Oris 2005), healthy lifestyles and behaviors (Abraído-Lanza et al. 2005; Lariscy et al. 2015), as well as in terms of selection effects (Oris and Alter 2001). With regard to the latter, most studies focus on the idea of a positive selection effect in the area of origin, in the sense that individuals who are young and healthy are more able and more likely to move than the sick, weak, elderly, and disabled (Khlat and Darmon 2003). For the nineteenth-century Belgian village of Sart, Oris and Alter (2001) observed, for example, that individuals from families that experienced death among their members during the previous two years were much less likely to out-migrate, compared with those from families in which everybody had survived.

Ever since the healthy migrant effect—initially referred to as an epidemiological paradox and later as the Hispanic or Latino paradox—was discovered among Latin American migrants in the US (Markides and Coreil 1986), scholars have doubted whether the results of such analyses are real, or if the healthy migrant effect is merely a statistical artifact resulting from measurement errors or biases toward healthy migrants. Not only are the results counterintuitive, and—at first glance—hard to reconcile with long-standing insights into health and mortality, but also the data on migrants are often of poorer quality than that of the native population (Razum 2006; Redstone Akresh and Frank 2008).

Doubts about the validity of the observed healthy migrant effect led to the formulation of the salmon bias hypothesis, which states that the observed lower mortality risks among migrants are the result of selective return-migration of the sick and elderly and those who are unable to adapt to and endure harsh working and living conditions (Deboosere and Gadeyne 2005). If migrants indeed have a tendency to go home before they die, their deaths do not contribute to the national death statistics in the country of study, but rather in the country of origin. If the second out-migration is not registered, this would lead to a situation in which migrants become “statistically immortal” in the society under study (Abraído-Lanza et al. 1999). Even if out-migration of the sick is registered, this can lead to measurement error since the presence of migrants in a society who do not die there is likely to lead to an inflated denominator, causing an artificially lowered mortality rate among migrants. These doubts suggest an overestimation of the healthy migrant effect, or even question the very existence of a health advantage of migrants.

## Objectives

In this paper we test the salmon bias hypothesis for *internal* migrants, both men and women, in Rotterdam during the latter half of the nineteenth and the early twentieth centuries by systematically comparing mortality risks among stayers and leavers, subdividing the latter category into returnees (migrants who return to their municipality of birth) and movers (migrants who moved to another destination than their municipality of birth). Given that women at the time had different migration patterns than men—they moved more often, but over shorter distances (Greefs and Winter 2016)—and faced diverging mortality risks in later life (Mourits 2017), we look at mortality risks not only for the entire study group, but also separately for women and for men.

Rotterdam was selected as a case study to test the salmon bias hypothesis for two main reasons. First in a previous study (Puschmann et al. 2016) comparing adult (ages 30+) mortality risks among migrants and natives in Antwerp, Rotterdam, and Stockholm (1850–1930), we found that the healthy migrant effect was particularly strong among internal migrants who moved to Rotterdam. We revealed that the health advantage of migrants in the port cities under study was related to, among others, the early life environment and positive selection effects. With respect to the latter, our previous findings showed an inverse relationship between migration distance and mortality risks. This led us to the conclusion that the most physically and mentally fit were more likely to migrate over long distances. Although we censored individual migrants upon out-migration, we wanted to test in a more systematic way whether selective return migration had biased the results of our event history analysis. This decision was strengthened by the fact that we found that certain subgroups of migrants in the population actually experienced excess mortality. In the city of Rotterdam this was the case for Italian and Italian-speaking Swiss immigrant men.

The second reason for choosing Rotterdam to test the salmon bias hypothesis is related to the nature of the data. The Historical Sample of the Netherlands (Mandemakers 2000) allows us to follow the life course of migrants (and natives) who left Rotterdam, at least as long as they moved within the national borders (97% of all internal migrants). Typically, the life courses of leavers are truncated upon out-migration in historical and contemporary datasets. Consequently, previous studies have, at best, only been able to estimate to what degree selective return-migration might have biased their results. Such estimations have led to contradictory and inconclusive results. Whereas some studies report that the healthy migrant effect is indeed caused by selective return-migration of the sick, weak, and elderly (Lu and Qin 2014), others found that this phenomenon only partially contributed to the observed effect (Khlat and Courbage 1996; Turra and Elo 2008), while still others reached the conclusion that it did not have an impact at all (Abraído-Lanza et al. 1999; Deboosere and Gadeyne 2005; Wallace and Kulu 2014).

Because the data allow us to track the final migration destination of internal migrants within the Netherlands, this case study can shed light on whether the salmon bias hypothesis can (partly) explain the mortality advantage of internal migrants we observed in our previous study (Puschmann et al. 2016).

## Research Setting

Like other European port cities at the time, Rotterdam received growing numbers of internal migrants during the latter half of the nineteenth and the early twentieth centuries. Thanks to urban in-migration and natural population growth, the population of Rotterdam increased from just over 90,000 inhabitants in 1850 to 332,000 in 1900, reaching 598,000 inhabitants by 1930. The majority of the internal migrants originated from the rural municipalities of the province of Zuid-Holland, which meant that the largest share of the newcomers in Rotterdam were peasants and agricultural laborers who were born in Rotterdam’s direct hinterland. The Dutch provinces of Noord-Brabant and Zeeland were also important sending areas (Van der Harst 2006). Among the urban immigrants in Rotterdam, women slightly outnumbered men (Puschmann 2015), and single women were particularly active as domestic servants (Bras 2003). Declining opportunities in the agricultural sector and the gradual destruction of the family economy were the main push factors for both men and women. The share of international migrants was stable at around 3% between 1850 and 1930 and consisted mainly of Germans, which is not surprising given the important trade relations with the German hinterland (Puschmann 2015).

Rotterdam’s attractiveness to migrants was mainly related to the growing employment opportunities in the port sector, industry, construction, and services. Thanks to the construction of the Nieuwe Waterweg—a direct connection between Rotterdam and the North Sea—and the high-speed industrialization of the German Ruhr and Rhine areas, Rotterdam swiftly turned into Europe’s largest port city. The port clearly functioned as an important pull factor for male internal and international migrants. By 1909, about 55% of the city’s working population was engaged in the port sector (Van de Laar 2000). The growth of the port went hand in hand with the revival of old industries and the advent of new industries, which usually concentrated on the handling of raw materials which arrived in the port. The construction of railways and tramways, as well as the introduction of busses, facilitated the migration of thousands of migrants but slowed down migration in the course of the first half of the twentieth century, as it allowed growing numbers of people to commute to Rotterdam (Puschmann 2015).

Bouman and Bouman (1955) showed that especially rural-to-urban migrants had a hard time integrating in Rotterdam. They put forward that newcomers were uprooted and ended up in a struggle for survival, as they landed in badly paid and dangerous jobs in the port and in construction, which made it difficult for them to make a living. Simultaneously they no longer could count on the social network in their home village, and it was difficult to create new social ties in Rotterdam, especially since the newcomers were—because of their dialect, low socioeconomic status, and different lifestyle—being looked down upon by the native Rotterdam population. More recent research has led to considerable changes to this picture. Internal migrants indeed entered the labor market at lower levels, but stayers were able to catch up with natives and even to outperform them in the long run (Puschmann 2015). Nevertheless, being born in the countryside was associated with substantially lower social status. Next, the facts that internal migrants in Rotterdam disproportionally stayed single, and that those who married did so on average at a later age, show that the social integration of internal migrants was indeed hampered, although this time rural-to-urban migrants were not disfavored relative to urban-to-urban migrants. Internal migrants in other port cities, including Antwerp and Stockholm, faced similar challenges (Puschmann et al. 2015).

## Methods

### Data

The data for the analyses was retrieved from the Data Set Life Courses Release 2010.01 from the Historical Sample of the Netherlands, a large historical demographic database with life course information on individuals born in the period 1812–1922 (Mandemakers 2000). The data are derived from the Dutch population registers as well as the vital registration of births, marriages, and deaths. The data collection began with a random sample of birth certificates, and the database makers aimed to “reconstruct” as many full life courses as possible. The *Data Set Life Courses Release* 2010.01 consists of 44,252 life courses, of which 62% are complete. Since the database managers have provided start and end dates for the periods in which the life course information of the research person is complete, we can determine the risk period for all individuals, with both full and partial life courses, in our survival models.

### Study Population

**Fig. 1:**
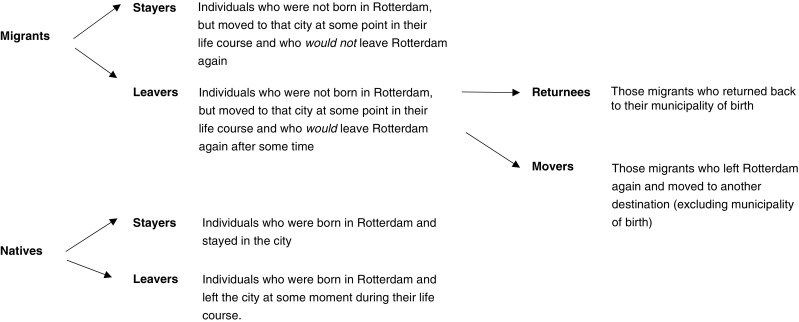
Overview of the different categories of migrants and natives in the analyses

We selected all individuals who lived in Rotterdam and its suburbs at some point between 1850 and 1940 (after their thirtieth birthday). This resulted in a dataset consisting of 1,452 research persons (756 natives and 696 migrants). Research persons who were born in Rotterdam are considered *natives*; individuals who were born elsewhere in the Netherlands and moved at some point to Rotterdam are *migrants*. The distinction between stayers, returnees, and movers is based on a combination of observed places of death, the declared destination of the last out-migration as it was specified in the population register of Rotterdam, and the place where a person was last recorded. The latter two criteria were only taken into account if the person was still alive at the end of the research period, or in case the place of death was unknown (*n* = 281). An overview of the classification of the research persons into natives, migrants, stayers, leavers, returnees, and movers is presented in Fig. 1.

For all individuals, all life course information from the database was retrieved, and individuals were only censored if they were still alive at the end of the study period or if they left the country. In our study group, 67% of all life courses are complete.

We included several fixed and time-varying variables in our analyses. The variable *migration status* is coded as native for those born in Rotterdam and migrant for those born outside of Rotterdam (but within the Netherlands). The variable *stayers/leavers* divides the study group into those who stayed in Rotterdam and those who left the city. *Age at arrival* notes the age that the migrant first arrived in the city and is divided into four categories: ages <15, 15–24, 25+, or unknown. In the analyses that include both men and women in the study group, we distinguish *sex* as women and men. Since birth dates spanned 60 years, we used *birth cohort* to categorize research persons into groups born 1850–1869, 1870–1889, and 1890–1910. Two variables were treated as time-varying, being updated from age 30 until the end of analysis time: *civil status* and *occupation*. *Civil status* was grouped into four categories: unmarried, married, separated/widowed, and unknown. *Occupation* is based on the HISCO codification (Van Leeuwen et al. 2002) and recoded into HISCLASS (Van Leeuwen and Maas 2011), and further categorized into four groups: professionals, foremen and skilled, day laborers and unskilled, and unknown. Finally, in order to identify the study population as natives, stayers, returnees, or movers, we included the variable *last destination*. This variable is first grouped as natives, migrants who stayed in Rotterdam, migrants who returned home, and migrants who migrated somewhere else. Later we further divided the native population into two groups: those who stayed and those who left.

### Statistical Analysis

We conduct survival analysis, first making use of Kaplan-Meier survival estimates to get an initial impression of the mortality differences for different categories of natives and migrants. In order to adjust for other factors, such as birth cohort, age at first in-migration, civil status, and occupation, we fit Gompertz proportional hazard models with all-cause mortality specified (ages 30+) as the failure event. The Gompertz model was chosen because it has been shown to fit adult human mortality well between ages 30 and 90 (Cleves et al. 2008). Further, on the basis of AIC/BIC testing criteria, the Gompertz model best fit our data (with the lowest AIC and BIC scores) compared with other parametric and semi-parametric models that were also tested (Cox proportional hazards, Weibull, and the exponential model).

By including the survival history of migrants upon departure, a more formal way of testing the salmon bias hypothesis than has been the case in previous studies is possible. In this way, we compare the mortality risks of three different groups of migrants—stayers, returnees, and movers—with that of the native population in order to determine if the observed healthy migrant effect was a result of selective out-migration. In the first analysis, we divide the migrant population into stayers and leavers. Next, we further divide the leavers into migrants who returned to their municipality of birth and migrants who moved to another destination. If we find that returnees have much higher mortality risks than the native population, we can confirm the salmon bias hypothesis. If our findings show, by contrast, no significant difference between natives and returnees or a lower mortality risk among the latter category we will reject the hypothesis and conclude that it is a “red herring.” Finally, we add a new element to the discussion by dividing the native population also into stayers and leavers. Too often, natives have been considered as a static category, even though a considerable share became migrants in the course of their lives. It is worth evaluating whether mortality risks also differed between natives who never left Rotterdam and natives who did leave since the healthy migrant effect suggests a self-selection mechanism in terms of health in the place of origin. Consequently, we should be able to find such a mechanism for their native counterparts. Our findings based on this distinction of separate categories of natives (those who stayed and those who left) are displayed in the Results.

We aimed for parsimonious models in order to maximize the statistical power for the newly added variables since our sample is relatively small. In order to benefit from the largest sample size possible, our main effects models are first presented for both sexes combined. We opt for nested models in which we include only those variables that improve the fit of the model, which are organized in a series of six models in which each additional variable was tested by use of log likelihood ratio tests. Based on these tests, two variables that we tested were not included in the analyses because they did not lead to a better fit: urban-rural birthplace and distance from birthplace. The final model, incorporating the main variables of interest and other controls from early and later life, leads to the best fit. See the Electronic Supplementary Materials (Table ESM-1) for descriptive statistics of all variables.

Next, we present the full models which we ran separately for men and women, given that there are sex differentials in mortality, in general, as well as that variables related to migration may differ for men and women—for example, propensities to migrate, reason to migrate, distance traveled, and ages at migration (cf. Greefs and Winter 2016; Greenwood 2008; Mourits 2017). These models were also designed as nested, but for simplification we present only the full models (the nested models are shown in Tables ESM-2 and 3). To more directly compare men and women with each other, we included interaction terms to measure how male and female natives and different groups of migrants differed in terms of mortality risk.

## Results

### Mortality Risks among Natives, Stayers, and Leavers

**Fig. 2:**
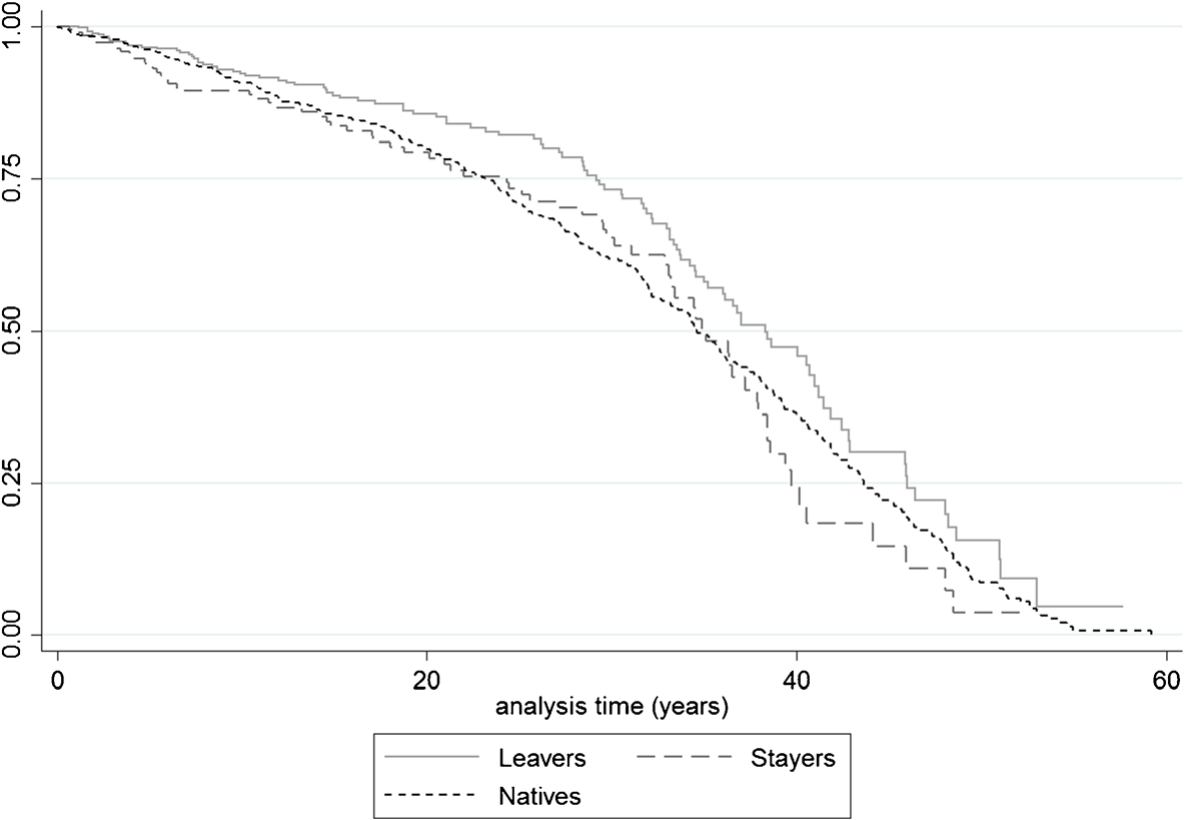
Kaplan-Meier survival estimates by stayers, leavers, and natives

Figure 2 shows the Kaplan-Meier survival estimates for natives and migrants, with the latter category subdivided into stayers and leavers. The graph shows that stayers have higher mortality risks than natives shortly after they enter the risk set, which might be related to the stress of first moving to an alien environment. However, between 25 and 35 years of analysis time the mortality risk of stayers is lower than that of the natives. From 37 years on their survival rates drop below those of the natives, suggesting that the stayers paid a long-term price for their move to the city. This might be related to the lack of a social network in a society with no national pension and health care system, in which the elderly and sick were dependent on care from their family, friends, and neighbors. However, the experience of the leavers is completely different. For the first 10 analysis years their mortality risks are comparable to those of natives, but subsequently their survival rates become considerably higher than those of the natives and the stayers. It seems therefore that this group was particularly healthy.

In Table 1, our nested models for both sexes are presented. Model I contains only the migration status variable. Unsurprisingly, migrants had a lower (though insignificant) risk of dying than natives (HR = 0.89; 95%; CI: 0.74–1.07). In Model II the stayer-leaver variable is included and the migration status variable becomes significant and its effect size strengthens (HR = 0.79; 95% CI: 0.63–0.99). Stayers have a 37% higher mortality risk compared with leavers, significant at the 5% level (95% CI: 1.02–1.85). In Model III age at in-migration is added. The migration status variable now loses its significance and the effect changes (HR: 1.02; 95% CI: 0.79–1.30), although the stayer-leaver variable stays significant and the effect remains stable. Migrants who arrived before their fifteenth birthday have a much lower risk of dying (HR: 0.26; 95% CI: 0.08–0.62) than the reference category of migrants who moved to Rotterdam after their twenty-fifth birthday, which is significant at the 5% level. The same is true for migrants who arrived between their fifteenth and twenty-fifth birthdays, but the effect size is considerably smaller (HR: 0.62; 95% CI: 0.35–1.07). Migrants who arrived at unknown ages had a significantly higher risk of dying (at the 10% level) compared with the reference category of migrants arriving at the ages of 25+ (HR: 0.62; 95% CI: 0.97–1.90). Sex is adjusted for in Model IV, which has no major influence on the other variables (only unknown age at arrival becomes more significant). In Model V we adjust for birth cohort, significant at the 0.01% level, which suggests an increase in the mortality risk for each successive cohort, most likely related to industrialization. In Model VI the time-varying covariates civil status and occupation were added to the models. These adjustments led to a stronger healthy migrant effect (although the variable stays insignificant) and an increase in the hazard ratio of the stayers, now with 83% higher mortality risk than leavers. The effects for age at arrival weakened, and the category of ages 15–24 becomes insignificant. The unknown age category, however, becomes stronger and highly significant. As it turns out, singles had a higher mortality risk than the reference category of married people (HR: 1.58; 95% CI: 1.18–2.09), and widowed and separated individuals also had a higher mortality risk (HR: 1.99; 95% CI: 1.42–2.66). An even stronger effect was found for individuals with unknown marital status (HR: 3.01; 95% CI: 2.37–3.81). For occupation we found that the foremen and skilled workers had higher mortality risk than the reference category of professionals (HR: 1.30; 95% CI: 0.97–1.72).

**Table 1:** Hazard ratios and confidence intervals for deaths at ages 30+ for men and women presented in nested models, Rotterdam (subjects = 1452; failures = 481)

	Model I		Model II		Model III		Model IV		Model V		Model VI	
	HR	CI	HR	CI	HR	CI	HR	CI	HR	CI	HR	CI
Migration status												
Native (ref)												
Migrant	0.89	[0.74–1.07]	0.79*	[0.63–0.99]	1.02	[0.79–1.30]	1.02	[0.79–1.30]	1.02	[0.79–1.30]	0.81	[0.63–1.04]
Stayers/leavers (migrants only)												
Leavers (ref)												
Stayers			1.37*	[1.02–1.85]	1.38*	[1.02–1.86]	1.39*	[1.03–1.88]	1.40*	[1.03–1.88]	1.83***	[1.34–2.50]
Age at arrival												
< 15					0.26*	[0.08–0.84]	0.26*	[0.08–0.84]	0.23*	[0.07–0.74]	0.36+	[0.11–1.16]
15–24					0.62+	[0.35–1.07]	0.62*	[0.36–1.08]	0.59+	[0.33–1.01]	0.77	[0.44–1.35]
25+ (ref)												
Unknown					1.36+	[0.97–1.90]	1.38*	[0.98–1.92]	1.46*	[1.04–2.03]	1.88***	[1.33–2.65]
Sex												
Women (ref)												
Men							1.16	[0.96–1.40]	1.11	[0.91–1.34]	1.22+	[0.98–1.51]
Birth cohort												
1850–1869 (ref)												
1870–1889									1.59***	[1.27–1.98]	1.86***	[1.48–2.33]
1890–1910									2.39***	[1.56–3.64]	3.02***	[1.96–4.64]
Civil status (time–varying)												
Unmarried											1.58**	[1.18–2.09]
Married (ref)												
Widowed / separated											1.99***	[1.49–2.66]
Unknown											3.01***	[2.37–3.81]
Occupation (time–varying)												
Professionals (ref)												
Foremen and skilled											1.40*	[1.04–1.88]
Day laborers and unskilled											1.28	[0.88–1.85]
Unknown											1.54**	[1.14–2.06]
log likelihood	−272.93		−270.79		−259.86		−258.69		−246.97		−198.87	

Controlled for age

Exponentiated coefficients and confidence intervals in brackets

+ *p* < 0.10, * *p* < 0.05, ** *p* < 0.01, *** *p* < 0.001

Given that migration variables could have a different relationship with adult mortality for men and women, Table 2 shows the full models separately by sex. A first observation is that, apart from the marital status variable, the effects are in the same direction, but less strong for men than for women, and the results are considerably more often significant among women. The latter is most likely related to the somewhat smaller sample size and the fewer number of failures (deaths) among men.

**Table 2:** Hazard ratios and confidence intervals for deaths at ages 30+ for women and men presented in separate models, Rotterdam

	Women		Men	
	HR	CI	HR	CI
Migration status				
Native (ref)				
Migrant	0.754+	[0.54–1.05]	0.860	[0.57–1.31]
Stayers/leavers (migrants only)				
Leavers (ref)				
Stayers	2.302***	[1.58–3.36]	1.370	[0.75–2.51]
Age at arrival				
< 15	0.186+	[0.03–1.37]	0.762	[0.17–3.47]
15–24	0.835	[0.44–1.59]	0.709	[0.23–2.17]
25+ (ref)				
Unknown	1.620*	[1.08–2.44]	2.550**	[1.34–4.86]
Birth cohort				
1850–1869 (ref)				
1870–1889	2.064***	[1.54–2.76]	1.719**	[1.20–2.47]
1890–1910	3.601***	[2.04–6.36]	2.642**	[1.36–5.12]
Civil status (time–varying)				
Unmarried	1.548*	[1.05–2.27]	1.618*	[1.05–2.49]
Married (ref)				
Widowed / separated	2.364***	[1.70–3.29]	0.868	[0.33–2.25]
Unknown	3.552***	[2.60–4.85]	2.600***	[1.78–3.79]
Occupation (time–varying)				
Professionals (ref)				
Foremen and skilled	1.459	[0.92–2.32]	1.26	[0.84–1.88]
Day laborers and unskilled	1.304	[0.72–2.38]	1.27	[0.79–2.06]
Unknown	1.578*	[1.02–2.44]	1.405	[0.87–2.28]
Subjects	969		680	
Failures	314		167	
log likelihood	−99.95		−85.03	

Controlled for age

Exponentiated coefficients and confidence intervals in brackets

+ *p* < 0.10, * *p* < 0.05, ** *p* < 0.01, *** *p* < 0.001

Only for women do we find a significant mortality advantage for the migrants compared with the reference category of natives (HR: 0.75; 95% CI: 0.54–1.05). For men the HR is 0.86, but not significant (95% CI: 0.57–1.31). Among the women the stayers had a 2.3 times higher mortality risk than the leavers, significant at the 0.001 level (95% CI: 1.58–3.56). Among the men the effect was in the same direction, but weaker and not significant (HR: 1.37; CI: 0.75–2.51). The younger the migrant women and men were when they arrived in the city, the lower their mortality risks were. Although this effect was only significant for women in the age category <15 (at the 0.1% level), this finding is particularly strong (with a HR of 0.19) relative to the reference category of ages 25+. For both men and women, migrants who arrived at an unknown age had a much higher (and highly statistically significant) mortality risk. Next, for men and women we observe significantly higher mortality risks for the cohort 1870–1889 and 1890–1910 compared with the reference cohort of 1850–1869. Unmarried men and women had higher mortality risks than married individuals. Widowed and separated women had a highly significant higher mortality risk compared with married women (HR: 2.35; 95% CI: 1.70–3.29). Additionally, men and women with an unknown marital status had a significantly higher mortality risk compared with the reference categories of married men and married women. Regarding occupation, we found significant effects only among women with an unknown occupation. They had a higher mortality risk compared with the reference category of professionals (HR: 1.58; 95% CI: 1.02–2.44).

### Mortality Risks among Natives, Stayers, Returnees, and Movers

**Fig. 3:**
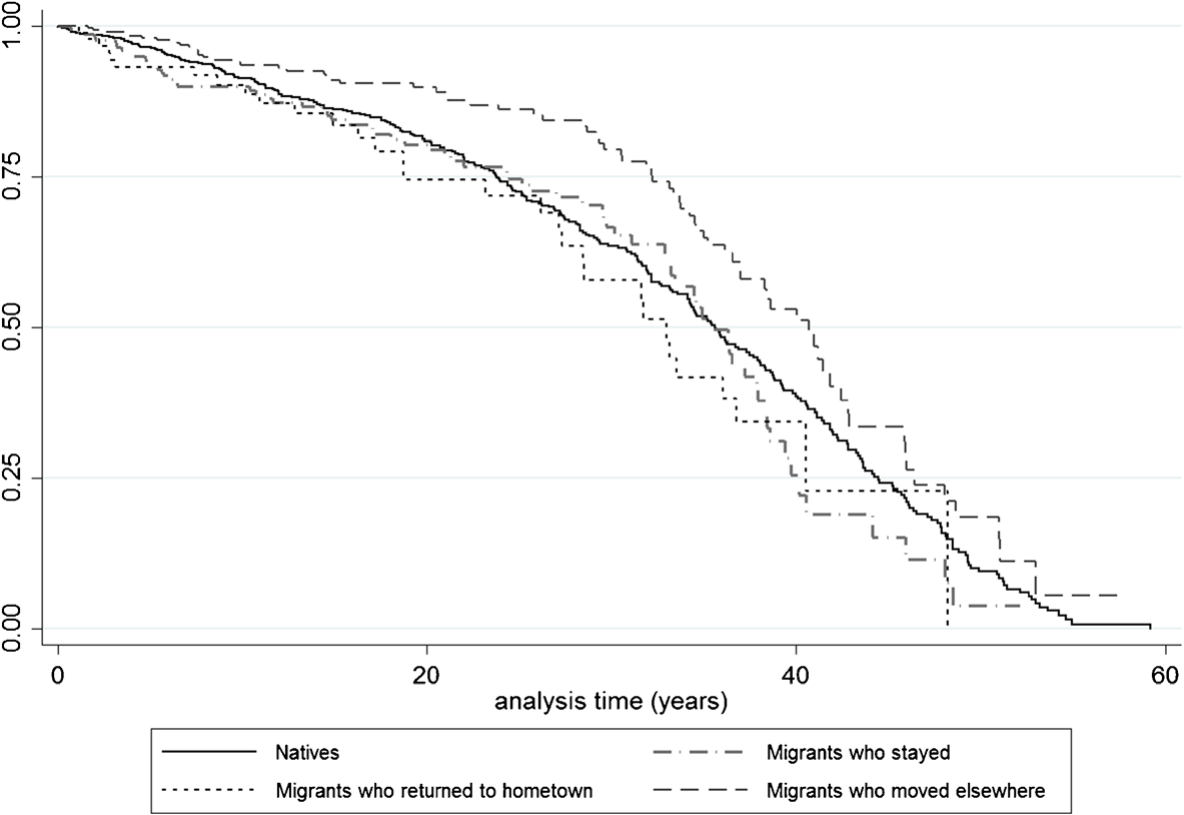
Kaplan-Meier survival estimates by last destination

Figure 3 shows the Kaplan-Meier curves for natives, migrants who stayed in Rotterdam, movers, and return migrants. The curves show that movers had much lower mortality risks than natives and migrants who stayed in Rotterdam. The survival rates of return migrants were somewhat below that of natives and migrants who stayed in Rotterdam, but the difference was not as pronounced as one would expect on the basis of the salmon bias hypothesis. After 20 years of analysis time the survival estimates become less reliable owing to small sample size. Judging on the basis of these K-M curves, the healthy migrant effect is caused by the group of movers, who have much lower mortality risks than all other groups.

Next, Table 3 shows the three fully adjusted Gompertz models for both sexes and for women and men separately. In the first model (both sexes combined), migrants who stayed in Rotterdam had a significantly higher mortality risk than the native population (HR: 1.67; 95% CI: 1.18–2.21). However, no significant difference in the mortality risks between natives and return migrants was found, and the effect size is so small that it cannot account for any healthy migrant effect (HR: 1.13; 95% CI: 0.72–1.98). Movers, by contrast, had a considerably lower mortality risk than the native population (HR: 0.76; 95% CI: 0.59–1.00) and was highly significant. For the women we observe the same results, but the effects for stayers (HR: 1.85; 95% CI: 1.29–2.66) and movers (HR: 0.71; 95% CI: 0.50–1.00) were stronger, while the effect size for the returnees was even smaller. In the model including only men, no significant results were found.

**Table 3:** Hazard ratios and confidence intervals for deaths at ages 30+ by last destination for men and women, Rotterdam (subjects: 1452; failures = 481)

	Both sexes		Women only		Men only	
	HR	CI	HR	CI	HR	CI
Natives (ref)	1		1		1	
Migrants who stayed in Rotterdam	1.67**	[1.18–2.21]	1.85**	[1.29–2.66]	1.163	[0.58–2.33]
Migrants who returned to home town	1.13	[0.72–1.98]	1.05	[0.54–2.05]	1.416	[0.63–3.18]
Migrants who moved elsewhere	0.76*	[0.59–0.99]	0.71*	[0.50–1.00]	0.821	[0.53–1.26]

Controlled for age, age at arrival, birth cohort, and time-varying civil status and time-varying occupation

Sex is controlled for in the model including both men and women

Exponentiated coefficients and confidence intervals in brackets

+ *p* < 0.10, * *p* < 0.05, ** *p* < 0.01, *** *p* < 0.001

**Fig. 4:**
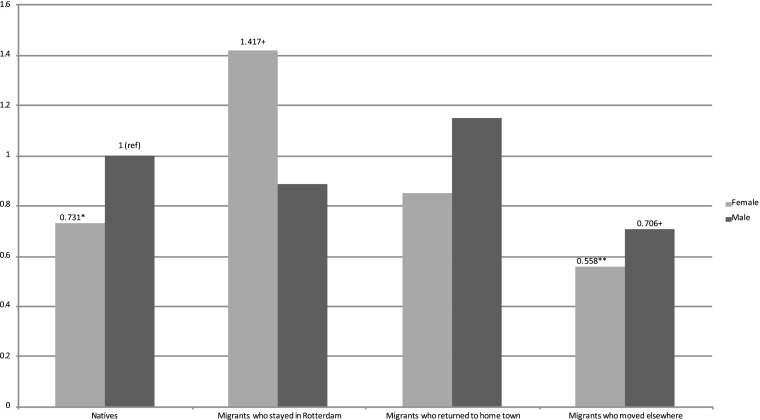
Relative mortality risks by sex and last destination (fully controlled; significant results noted)

Extending this analysis, in order to compare both men and women among natives, stayers, returnees, and movers, we ran a model including interaction terms for sex and the last destination variable (Fig. 4). Compared with native men, only returnees had elevated mortality risks, with about 15% higher mortality, but the result was not significant (HR: 1.15; 95% CI: 0.52–2.50). Both stayers (HR: 0.83) and movers (HR: 0.70) had lower mortality risks, with the latter category significant at the 10% level. For women, relative to the reference category of native men, there were significantly lower mortality risks for natives (HR: 0.73; 95% CI: 0.56–0.94) and for movers (HR: 0.56; 95% CI: 0.38–0.80). Female returnees also had lower mortality risks, though not statistically significant (HR: 0.85; 95% CI: 0.43–1.65). The only female group with excess mortality was found for stayers, with just over 40% higher mortality risk compared with native men, significant at the 10% level (HR: 1.42; 95% CI: 0.98–2.04). The latter result is quite striking. It suggests that women who stayed in Rotterdam were a less favorable selection of the population of origin in terms of health and human capital, and/or that they paid a higher health price for their migration. The former is underlined by a recent study by Hilde Greefs and Anne Winter, in which they showed that women who moved over a longer distance to Antwerp in the latter half of the nineteenth century were—contrary to men—more often from a more modest background (Greefs and Winter 2016). At the same time it is not unthinkable that women—because of their limited human capital—became marginalized upon arrival in Rotterdam. Others with more means might have moved on, whereas those who stayed in contact with their family of origin and the community in which they grew up might have returned after domestic service.

### Comparing Mortality Risks of Stayers and Leavers for both Migrants and Natives

**Fig. 5:**
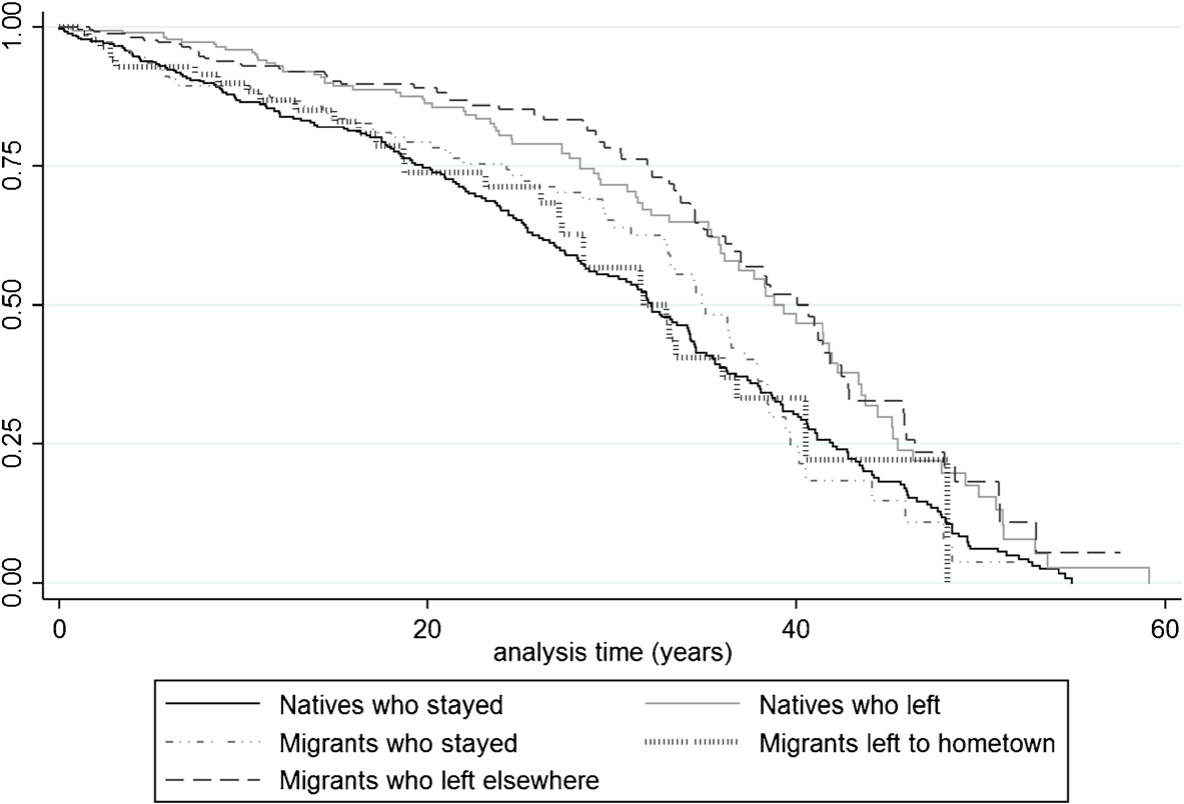
Kaplan-Meier survival estimates by last destination

Finally, we divide the native population also into stayers and leavers. The Kaplan-Meier curves in Fig. 5 show that natives who left Rotterdam had higher survival probabilities than natives who stayed. The K-M curve of the leaving natives is similar to that of migrants who moved to another destination in the Netherlands. However, between 15 and 35 years of analysis time, the survival probabilities of the leaving natives were even lower than that of the moving migrants.

**Fig. 6:**
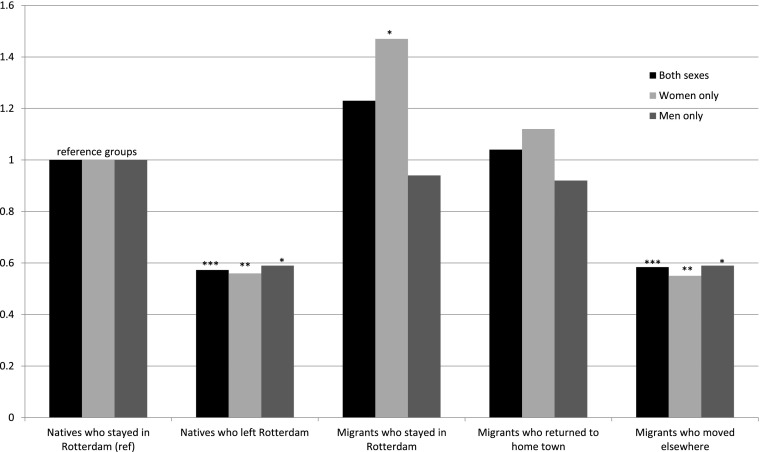
Relative mortality risks by last destination for both sexes, women and men separately (controlled for age, age at arrival, birth cohort, and time-varying civil status and occupation) + *p* < 0.10, * *p* < 0.05, ** *p* < 0.01, *** *p* < 0.001

Next, Fig. 6 shows the Gompertz model with the distinction between stayers and leavers among the native population in three models: both sexes combined, only women, and only men. The models are adjusted for age, age at arrival, sex (in the combined model), birth cohort, and time-varying civil status and occupation. Compared with the reference category of native stayers, natives who left Rotterdam have highly significant relative mortality risks at just under HR = 0.6 across all models. Similarly, nearly the same results, in terms of strength and significance, were found for migrants who left for another destination. No significant differences were found for return migrants relative to the reference category in all three models. Overall, men and women had similar results with the exception of migrants who stayed in Rotterdam. Female migrants who stayed had around 47% higher relative mortality risk than female natives, significant at the 5% level (HR: 1.47).

## Discussion, Policy Implications, and Future Research

The analyses in this paper show that we can reject the salmon bias hypothesis for our specific case study. Even though we found some elevated mortality risk for male returnees at first glance, the observed lower mortality among internal migrants in Rotterdam are real and were not caused by selective return migration of the sick, weak, and elderly because no significant difference in mortality risks between returnees and the native population was found. The salmon bias hypothesis is thus a red herring in the case of late-nineteenth- and early-twentieth-century Rotterdam. Movers experienced the lowest relative mortality risks of the entire population under study. If we factor in that natives who left Rotterdam also had significantly lower mortality risks than natives who stayed in the Dutch port city, we can only conclude that migration and good health are even more strongly correlated than we could imagine on the basis of the previous studies: The healthier people were, the more they moved, and this was true for both men and women, who experienced similar effects in terms of their migration patterns.

Although men and women experienced similar effects overall, we further investigated sex differences since the motivation to move and the patterns of migration differed between men and women. We find one significant distinction in our analyses between men and women. Female migrants who stayed in Rotterdam had a higher mortality risk (than female natives of Rotterdam), but men did not (they had slightly lower mortality risk compared with male natives). This seems to suggest that for migrant women the selection effect was not as strong as it was for men; the difference between migrant women and those who stayed in their region of origin was not as great (cf. Greefs and Winter 2016). Perhaps because of their limited human capital or the lack of a social network, they might have ended up in trouble in the city, which could have prevented them from returning home or moving to another destination. One might think about domestic servants who gave birth to an illegitimate child or about women who ended up in prostitution. The literature on migration in this era suggests indeed that female migrants were disproportionally engaged in out-of-wedlock fertility and prostitution (Fuchs and Moch 1990; Moch 2003). However, in the case of Rotterdam such claims would require further investigation.

The fact that we did not find a salmon bias effect in our data does not mean that this result can be automatically extrapolated to other populations in other times and regions. As always, the historical context in which human behavior is being shaped has to be taken into account. Certain migrant populations might be more inclined to move to their home region once they fall seriously ill, and this could in fact lead to a real salmon bias effect. It is therefore crucial to replicate formal tests of the salmon bias hypothesis for other populations, and to study migration and mortality patterns against the background of the societies migrants came from as well as the societies they moved to. This means that future research would do well to take the culture, religion, traditions, and family systems of the migrant populations under study into account.

The results of this study also have an important implication for contemporary health policy. The healthy migrant effect suggests that all migrant groups fare better than the native population, but this is only true for those migrants who are most mobile. The effect, therefore, likely underestimates the health problems among migrants who live in a receiving society for a longer period of time. The fact that stayers fare much worse than movers also suggests that migrants pay a health price for adaptation. In light of our findings, future studies on migrant health should distinguish between stayers and leavers and, within those groups, between men and women.

## Electronic supplementary material

ESM 1 (PDF 177 kb)
