# Supplementary material for: Salmon Bias or Red Herring? Comparing Adult Mortality Risks (Ages 30–90) between Natives and Internal Migrants: Stayers, Returnees and Movers in Rotterdam, the Netherlands, 1850–1940
Source: Hum Nat. 2017 Oct 17;28(4):481–99. doi: 10.1007/s12110-017-9303-1 (PMC5662680; doi:10.1007/s12110-017-9303-1)
Supplement: Supplementary file 1 — (PDF 177 kb) [file 12110_2017_9303_MOESM1_ESM.pdf]

Electronic Supplementary Material (ESM)

for *Human Nature* 28(4), 2017, http://dx.doi.org/10.1007/s12110-017-9303-1

Salmon Bias or Red Herring?
Comparing Adult Mortality Risks (Ages 30–90) between Natives and Internal Migrants: Stayers, Returnees and Movers in Rotterdam, the Netherlands, 1850–1940

Paul Puschmann, Robyn Donrovich, and Koen Matthijs

Email: P.Puschmann@let.ru.nl

Table ESM-1 Descriptive statistics for the study group of men and women in Rotterdam (*n*=1,452)

Table ESM-2. Hazard ratios and confidence intervals for deaths at ages 30+ for women presented in nested models, Rotterdam (subjects=969; failures=314)

Table ESM-3. Hazard ratios and confidence intervals for deaths at ages 30+ for men presented in nested models, Rotterdam (subjects=680; failures=167)

Table ESM-1 Descriptive statistics for the study group of men and women in Rotterdam (*n*=1,452)

Variable	Categories	Cases	% Distribution	Deaths
**Migration status**	Native	756	52.1	303
	Migrant	696	47.9	178
**Stayers/leavers**	Leavers	464	32.0	106
	Stayers	232	16	72
	Natives	756	52.1	303
**Last destination**	Natives who stayed in Rotterdam	409	28.2	217
	Natives who left Rotterdam	347	23.9	86
	Migrants who stayed in Rotterdam	232	16	72
	Migrants who emigrated home	103	7.1	31
	Migrants who emigrated elsewhere	361	24.9	75
**Age at arrival**	<15	83	5.7	3
	15-24	151	10.4	17
	25+	194	13.4	52
	Unknown, N/A	1024	70.5	409
**Sex**	Women	902	62.1	314
	Men	550	37.9	167
**Birth cohort**	1850-1869	447	30.8	299
	1870-1889	552	38	151
	1890-1910	453	31.2	31
**Civil status (time-varying)**	Unmarried	356	24.5	78
	Married	606	41.5	136
	Widowed / separated	208	14.3	94
	Unknown	282	19.7	173
**Occupation (time-varying)**	Professionals	287	19.7	64
	Foremen and skilled	454	31.3	150
	Day laborers and unskilled	143	9.9	50
	Unknown	568	39.1	217
**Total**		**1,452**	**100%**	**481**

Note: Percentages for distributions rounded

Table ESM-2. Hazard ratios and confidence intervals for deaths at ages 30+ for women presented in nested models, Rotterdam (subjects=969; failures=314)

		MODEL I		MODEL II		MODEL III		MODEL IV		MODEL V	
		H.R.	C.I.	H.R.	C.I.	H.R.	C.I.	H.R.	C.I.	H.R	C.I.
Migration status	Native (ref)										
	Migrant	0.96	[0.76-1.21]	0.81	[0.60-1.06]	1.02	[0.71-1.34]	0.96	[0.70-1.33]	0.75+	[0.54-1.05]
Stayers/leavers (migrants only)	Leavers (ref)										
	Stayers			1.58*	[1.10-2.27]	1.51*	[1.05-2.17]	1.52*	[1.06-2.19]	2.30***	[1.58-3.36]
Age at arrival	<15					0.13*	[0.02-0.96]	0.11*	[0.02-0.80]	0.19+	[0.03-1.37]
	15-24					0.67	[0.35-1.26]	0.62	[0.33-1.18]	0.84	[0.44-1.59]
	25+ (ref)										
	Unknown					1.16	[0.77-1.72]	1.20	[0.80-1.78]	1.62*	[1.08-2.44]
Birth cohort	1850-1869 (ref)										
	1870-1889							1.66***	[1.25-2.21]	2.06***	[1.54-2.76]
	1890-1910							2.68***	[1.53-4.68]	3.60***	[2.04-6.36]
Civil status (time-varying)	Unmarried									1.55*	[1.05-2.27]
	Married (ref)										
	Widowed / separated									2.36***	[1.70-3.29]
	Unknown									3.55***	[2.60-4.85]
Occupation (time-varying)	Professionals (ref)										
	Foremen and skilled									1.46	[0.92-2.32]
	Day laborers and unskilled									1.30	[0.72-2.38]
	Unknown									1.58*	[1.02-2.44]
log likelihood		−161.64		−158.6		−152.266		−143.51		−105.43	

Controlled for age

Exponentiated coefficients and confidence intervals in brackets

+ p < 0.10, * p < 0.05, ** p < 0.01, *** p < 0.001

Table ESM-3. Hazard ratios and confidence intervals for deaths at ages 30+ for men presented in nested models, Rotterdam (subjects=680; failures=167)

		MODEL I		MODEL II		MODEL III		MODEL IV		MODEL V	
		H.R.	C.I.	H.R.	C.I.	H.R.	C.I.	H.R.	C.I.	H.R	C.I.
Migration status	Native (ref)										
	Migrant	0.78	[0.56-1.07]	0.77	[0.54-1.11]	1.08	[0.73-1.61]	1.09	[0.73-1.62]	0.86	[0.57-1.31]
Stayers/leavers (migrants only)	Leavers (ref)										
	Stayers			1.01	[0.58-1.76]	1.27	[0.71-2.27]	1.27	[0.71-2.27]	1.37	[0.75-2.51]
Age at arrival	<15					0.56	[0.13-2.47]	0.52	[0.12-2.32]	0.76	[0.17-3.47]
	15-24					0.53	[0.18-1.60]	0.50	[0.17-1.52]	0.71	[0.23-2.17]
	25+ (ref)										
	Unknown					1.94*	[1.05-3.59]	2.11*	[1.14-3.93]	2.55**	[1.34-4.86]
Birth cohort	1850-1869 (ref)										
	1870-1889							1.52*	[1.06-2.18]	1.72**	[1.20-2.47]
	1890-1910							2.15*	[1.11-4.12]	2.64**	[1.36-5.12]
Civil status (time-varying)	Unmarried									1.62*	[1.05-2.49]
	Married (ref)										
	Widowed / separated									0.87	[0.33-2.25]
	Unknown									2.60***	[1.78-3.79]
Occupation (time-varying)	Professionals (ref)										
	Foremen and skilled									1.26	[0.85-1.88]
	Day laborers and unskilled									1.27	[0.79-2.06]
	Unknown									1.41	[0.87-2.28]
log likelihood		−109.67		−109.67		−103.69		−99.95		−85.03	

Controlled for age

Exponentiated coefficients and confidence intervals in brackets

+ p < 0.10, * p < 0.05, ** p < 0.01, *** p < 0.001
